# International perspective on common core competencies for occupational physicians: a modified Delphi study

**DOI:** 10.1136/oemed-2015-103285

**Published:** 2016-04-13

**Authors:** Drushca Lalloo, Evangelia Demou, Sibel Kiran, Marianne Cloeren, René Mendes, Ewan B Macdonald

**Affiliations:** 1 Healthy Working Lives Group, Institute of Health and Wellbeing, College of Medical, Veterinary and Life Sciences, University of Glasgow, Glasgow, UK; 2 MRC/CSO Social and Public Health Sciences Unit, Institute of Health and Wellbeing, College of Medical, Veterinary and Life Sciences, University of Glasgow, Glasgow, UK; 3 Department of Occupational Health and Medicine, Hacettepe University, Institute of Public Health, Sihhiye-Ankara, Turkey; 4 Managed Care Advisors, Inc., Bethesda, Maryland, USA; 5 National Association of Occupational Medicine (ANAMT/Brazil), São Paulo, Brazil

**Keywords:** competencies, occupational physician training, Delphi study, occupational medicine

## Abstract

**Objectives:**

The competencies required of occupational physicians (OPs) have been the subject of peer-reviewed research in Europe and individual countries around the world. In the European Union (EU), there has been development of guidance on training and common competencies, but little research has extended beyond this. The aim of this study was to obtain consensus on and identify the common core competencies required of OPs around the world.

**Methods:**

A modified Delphi study was carried out among representative organisations and networks of OPs in a range of countries around the world. It was conducted in 2 rounds using a questionnaire based on the specialist training syllabus of a number of countries, expert panel reviews and conference discussions.

**Results:**

Responses were received from 51 countries around the world, with the majority from Europe (60%; 59%) and North and South America (24%; 32%) in rounds 1 and 2, respectively. General principles of assessment and management of occupational hazards to health and good clinical care were jointly considered most important in ranking when compared with the other topic areas. Assessment of disability and fitness for work, communication skills and legal and ethical issues completed the top five. In both rounds, research methods and teaching and educational supervision were considered least important.

**Conclusions:**

This study has established the current priorities among OPs across 51 countries of the common competencies required for occupational health (OH) practice. These findings can serve as a platform for the development of common core competencies/qualifications within specific geographical regions or internationally. This is particularly pertinent with globalisation of commerce and free movement within the EU.

**What this paper adds**

- Earlier studies have identified common core competencies for OPs in Europe and have examined the professional development of key OH professionals around the world. These were undertaken over a decade ago and with the rapid evolution of OH practice, training and competencies require regular review and update.
- This study has established current priorities among specialist OPs internationally of the common competencies required for OH practice.
- General principles of assessment and management of occupational hazards to health and good clinical care were jointly considered most important in ranking. Research methods and teaching and educational supervision were considered least important.
- These up-to-date and mutually identified priorities can serve as a platform for the development of local training curricula and common core competencies/qualifications within specific geographical regions or, indeed, internationally. They can also help to inform global policy on the delivery of OH services and, importantly, quality standards.

## Introduction

Occupational health (OH) practice is evolving around the world from the traditional considerations of protection from work hazards, fitness for work and work injury care to include management of the health and well-being of the working population.^1^

The role of occupational physicians (OPs) historically has varied among countries depending on national legislation,^1^ employer, employee and workforce needs.^2^ There are differences in models of delivery with increasing use of a multidisciplinary healthcare approach in some countries. Although similar in some aspects, OH differs from other medical practice settings in that it is framed by additional legal, ethical and regulatory requirements.^3^ The modern specialist OP is faced with the challenge of incorporating the evidence base, the recommended best practice, ethical guidance and legislative requirements into day-to-day clinical practice, and often within time constraints.^3^

Recent decades have seen a decline in the traditional industrial diseases with the emergence of a range of health conditions reflecting advances in technology and the changing workplace. There is increasing awareness of the important role of the biopsychosocial approach.^4^ ^5^ An increasing focus on health promotion is evident as compared to the historical emphasis on reduction of occupational disease. Sickness absence management,^6^ vocational rehabilitation^7^ and management of the ageing worker^8–10^ have emerged as newer areas of practice. Clinical leadership and management skills are becoming important, even for OPs not in formal management roles, with the expanding multidisciplinary nature of the specialty. This evolution of OH practice has brought with it fresh challenges and changing priorities.

Although OH practice can vary among countries, there are core values, knowledge and skills characterising the specialty.^1^ The competencies required of occupational medical practitioners have been the subject of peer-reviewed research in individual countries around the world.^11–16^ A study on requirements for occupational medicine (OM) training in Europe identified that respondents had traditional disease-focused views of the competencies required and that competencies were lagging behind the evolving definition of OH.^13^ A study on the customer perspective identified substantial differences in rating and ranking of the relative importance of competencies between OPs and their customers (employers, employees and their representatives), with competency in law and ethics being the highest priority for these customers.^15^ A global survey examining the professional development and distinguishing features of OH professionals (OPs, OH nurses, hygienists and ergonomists) around the world identified that OPs had higher scores for the administrative/management skills compared with all other professionals.^11^ This included health and safety considerations as well as knowledge of relevant policies, regulations and law. In terms of curricula, biostatistics, fundamentals of OH, toxicology, epidemiology and industrial hygiene were the courses most frequently identified for physicians.^11^ Although limited to a degree by the response rate, there was general consensus among the respondents about the work roles of OH professionals.^11^ A systematic review of the literature has highlighted that there is currently a limited evidence base on which to develop common competencies for the future training needs in OM.^12^

Specialist training competency requirements for OM have been developed in a number of countries including the UK,^17^ the USA,^18^ Australia^19^ and Brazil.^20^ As an outcome of the European study,^13^ the WHO^14^ and the Occupational Medicine Section of the European Union of Medical Specialists (UEMS)^16^ have developed guidance on training and competencies in Occupational Medicine in Europe and at European Union (EU) level to help achieve consistency of practice and with the increasingly common occupational health and safety EU Framework directives.

The aim of this study was to obtain consensus on and identify the common core competencies required of OPs around the world by using a modified Delphi technique.

The Delphi method (aka Delphi technique) is widely used in social sciences research to solicit the opinions of experts through a series of questionnaires. It is one of the most common methodologies used to identify priorities in OH.^13^ ^15^ ^21–23^ It has been widely used to establish OH research priorities in different countries and among a range of stakeholders.^21–25^ It has also been used to identify priorities for understanding and managing occupational allergy.^26^ Modified versions were used in the previous European and customer perspective reviews on required OP competencies.^13^ ^15^ The method itself^27^ comprises a series of questionnaires starting with open-ended broad questions and concluding when consensus has been established at a sufficient level on the key priorities. A review of the literature has not identified any study seeking consensus on core competency requirements for OPs internationally.

## Methods

**Figure 1:**
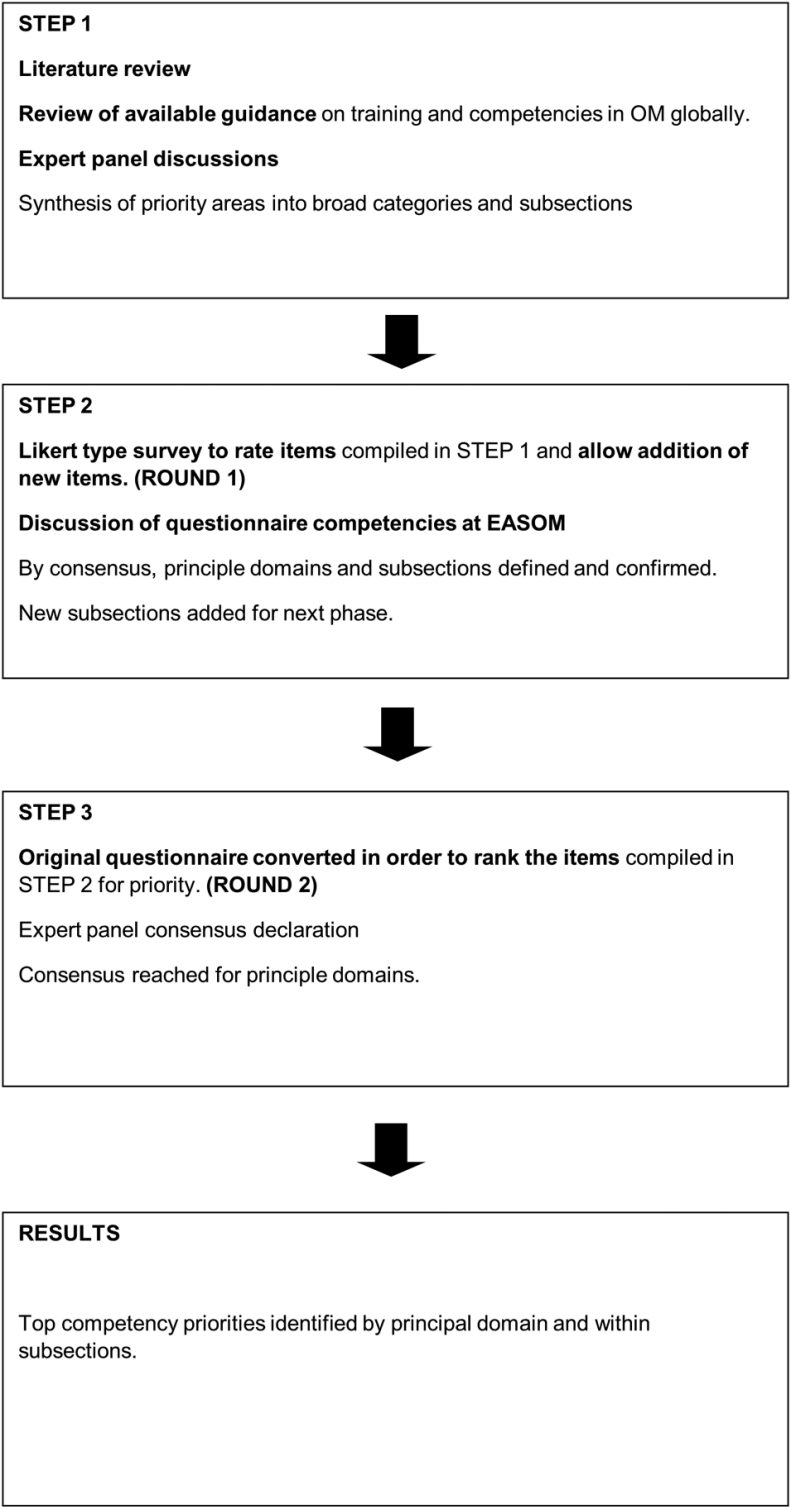
The modified Delphi process described.

A modified Delphi study was carried out among representative networks of OPs in a range of countries around the world. The method used is illustrated in figure 1.

### Step 1

A literature review was carried out and available guidelines on training competencies for OM were identified and reviewed. A series of expert panel discussions followed face to face and by email correspondence, to consider current and emerging topics in the specialty. This included senior OPs covering the UK, Europe, North and South America and Australasia, as well as the author team. An initial questionnaire was developed based on these discussions and on the specialist training syllabus of a number of countries including the UK Faculty of Occupational Medicine,^17^ the Occupational Medicine Section of the UEMS guidance on training in OM at EU level,^16^ the American College of Occupational and Environmental Medicine's Occupational and Environmental Medicine Competencies—2014^18^ and the Australasian Faculty of Occupational and Environmental Medicine Training Curriculum.^19^ Emerging areas of practice identified within the speciality and from the literature, notably, the ageing worker,^8–10^ sickness absence management^6^ and vocational rehabilitation,^7^ were also included. We identified broad categories (principal domains) and key subsection items within these domains.

Contacts were established with key members from national societies of OM or representative organisations and networks of the specialty internationally and agreement gained to participate and disseminate the questionnaire within their respective societies and networks. These included the following:

- The European Association of Schools of Occupational Medicine (EASOM)
- The American College of Occupational and Environmental Medicine members (ACOEM)
- The Argentinian Federation of Occupational Medicine (FAMETRA)
- The Australasian Faculty of Occupational and Environmental Medicine (AFOEM)
- The Brazilian National Association of Occupational Medicine (ANAMT)
- The Colombian Society of Occupational Medicine (SCMT)
- The Faculty of Occupational Medicine, Ireland
- The Mexican National Federation of Occupational Health (FENASTAC)
- The Peruvian Society of Occupational Health (SOPESO)
- The UK Faculty of Occupational Medicine
- The UK Society of Occupational Medicine
- Occupational Medicine Section of the European Union of Medical Specialists (UEMS)

It was specified that for the purposes of this study, the questionnaire only be distributed to and completed by specialist/board-certified OPs. The survey was carried out in two rounds (see figure 1).

### Step 2: round 1 ‘rating’ questionnaire

The questionnaire comprised 12 principal domains covering the different topic areas of OH practice. Within these were subsection items detailing specific competencies pertaining to that domain. This ranged from one principal domain having as little as 3 subsections and another having as many as 11.

The initial questionnaire was circulated and respondents were asked to indicate the relative importance of the items. Open-ended questions were included, allowing respondents to add to the lists where appropriate. Respondents were asked to give each item on the list a separate score from 0 to 5. A score of 0 indicated that the item was not necessary, 1 indicated it was of minimal importance and 5 indicated it was most important or essential. The competency items were discussed at an international conference in Glasgow in August 2014 on the subject of specialist OM training competencies hosted by the European Association of Schools of Occupational Medicine (EASOM) inviting suggestions for competency items not already included.

### Step 3: round 2 ‘ranking’ questionnaire

On collation of the initial questionnaire responses, a second modified questionnaire was then produced, retaining the same 12 principal domains but including new subsection items derived from the first round open-ended question responses and the conference discussions. No items were removed from the lists. This second questionnaire was then circulated to the same key contacts as the first round and they were asked to distribute to their networks. Specialist/board-certified OPs that received the link were invited to participate irrespective of whether they had taken part in round 1 or not.

On this occasion, respondents were asked to place each of the items in rank order for the principal domains and their subsections. The item considered most important was given a rank of 1, next most important 2 and so on. It was not permitted to give two items in any given domain the same score. Additional items were not invited in this round, although a section for comments was included. Subsections were not presented in any particular order to avoid influencing respondents. Responses to the second questionnaire were analysed by averaging the rank orders to produce a mean score for each principal domain and for each subsection item within the given domains. As some domains had as many as 11 subsection items and some as few as 3, the mean scores were standardised to a 1–10 scale (see online supplementary material for complete list of average and standardised scores), to allow some comparison of the relative importance of subsection items in the different domains and overall. The standardised mean score also gives an indication of the consensus of opinion with the lowest scores indicating that many respondents gave this item high priority. High scores indicate that most respondents gave an item a low priority score. Subsection mean standardised scores were subsequently weighted using a scale from 1 to 12 based on the ranking order of their respective principal domains (ie, subsections within good clinical care had a weighting of 1 and subsections in research methods a weighting of 12) (table 3).

10.1136/oemed-2015-103285.supp1 Supplementary data

The questionnaires were circulated in English using a SMART survey link via electronic mail. Both were piloted in advance by an internationally representative sample of OPs, to validate their ease of use and comprehension in terms of language. A participant information sheet was included at the beginning of each survey and all participants were required to complete a consent agreement question, prior to proceeding with completion of the questionnaire. Job titles were coded by the researchers into three main job categories after assessment of the self-declared job titles and place of work provided by respondents. If respondents were solely involved in clinical OH practice, they were categorised as an ‘OP’, if they had a management title, they were labelled an ‘OP/manager’ and if they had an academic role, the ‘OP/academic’ category was applied. Areas of current OH practice comprised work in a healthcare setting—for example, a hospital (healthcare), public/private sector organisations (industry), participation in teaching or research (academic) or any work sector not covered by these (other). Respondents were asked to self-select their sector type and could choose more than one category. Intergroup (age, sex, years of experience, continent/region) comparisons of the group ranking were performed using the Spearman's rank test. Trainee responses were included as, being active in the process of training, their perspectives were deemed valuable. Furthermore, this was not a study of leaders in the field but of practising specialist OPs.

The first round questionnaire took place between June and August 2014 and the second between January 2015 and April 2015. Approximately 1 month after sending both questionnaires to participants by email, two reminder emails were sent to increase the response rate. The data were analysed using SPSS Statistics V.21 (SPSS. IBM SPSS Statistics for Windows. Armonk, New York, USA: IBM Corp, 2013). All statistical tests are based on the 95% confidence level.

## Results

### Round 1: rating

A total of 339 responses to the first questionnaire were received from 51 different countries around the world. The demographic distribution of responses is presented in table 1.

**Table 1:** Responses by age, sex, continents, job title and years of experience for rounds 1 and 2

	Round 1 (n=339)		Round 2 (n=232)	
Features	Frequency	Per cent	Frequency	Per cent
Age range category				
25–34	11	3.3	4	1.7
35–44	56	16.7	47	20.3
45–54	118	35.1	72	31.2
55–64	117	34.8	89	38.5
65–74	34	10.1	19	8.2
Total	336	100	231	100
Sex				
Male	213	63.4	150	64.9
Female	123	36.6	81	35.1
Total	336	100	231	100
Continents				
South and Central America	29	8.6	24	10.3
North America	52	15.3	51	22
Global	4	1.2	4	1.7
Europe	203	59.9	137	59.1
Australia and Oceania	21	6.2	8	3.4
Asia	26	7.7	7	3
Africa	4	1.2	1	0.4
Total	339	100	232	100
Job title				
OP	228	68.7	165	71.1
OP/manager	60	18.1	43	18.5
OP/academic	39	11.7	23	9.9
Trainee	5	1.5	1	0.4
Total	332	100	232	100
Years of experience	Mean±SD (min-max) n=339 19.6±10.1 (1–50)		Mean±SD (min-max) n=230 21±10.4 (1–50)	

OP, occupational physician.

All six continents were represented: Europe (59.9%), North America (15.3%), South and Central America (8.6%), Asia (7.7%), Australia and Oceania (6.2%) and Africa (1.2%), with 1.2% of OPs employed globally. The OPs were 63.4% male and 36.6% female. The majority of respondents (69.9%) were aged 45–64. The distribution by job category was 68.7% OP, 18.1% OP/manager, 11.7% OP/academic and 1.5% OP trainee. The mean value of years of expertise was 19.6±10.1, with a minimum of 1 and a maximum of 50.

The main area of practice was industry (44.8%), followed by healthcare services (40.7%) and academia (23.6%), although a notable degree of crossover was evident with OPs frequently working across a range of sectors.

Comparisons of the importance to respondents of the 12 principal domains are presented in table 2.

**Table 2:** Priorities in principal domains; rating—round 1 results

Rating score	Principal domains (n=339)	Yes (%)	No (%)	Not relevant (%)
1	General principles of assessment and management of occupational hazards to health	98.8	0.6	0.6
2	Communication skills	98.5	0.6	0.9
3	Ethical and legal issues	97.9	0.6	1.5
4	Team working and leadership skills	97.1	0.9	2.1
5	Assessment of disability and fitness for work	96.5	2.1	1.5
6	Health promotion	95	3.2	1.8
7	Good clinical care	94.7	2.1	3.2
8	Clinical governance/clinical improvement	94.1	2.7	3.2
9	Environmental issues related to work practice	92.9	4.7	2.4
10	Management skills	92.6	2.9	4.4
11	Teaching and educational supervision	90.6	5.3	4.1
12	Research methods	90.3	5.6	4.1

All the competency areas were regarded as important, with scores of 90% and over in every domain (no statistically significant difference among domains). General principles of assessment and management of occupational hazards to health scored most highly when compared with the other topic areas, followed by communication skills and ethical and legal issues. Conversely, research methods scored the lowest, followed by teaching and educational supervision. From this round, the new subsection items derived from the open-ended questions included the following: the principles of toxicology, ergonomics, occupational/industrial hygiene and travel medicine (within general principles of assessment and management of occupational hazards to health); motivational interviewing (within communication skills) and additional environmental health competencies.

### Round 2: ranking

No significant differences in the distributions of gender (Fisher's exact test p=0.483), age group (χ^2^ Yates: 5.096, p=0.278), job practice (χ^2^ Yates: 4.038, p=0.257) and years of experience (independent t test, t value=−1.185, p=0.236) were identified between the respondents of the first and second rounds. Round 2 results are displayed in table 3.

**Table 3:** Priorities in principal domains; ranking—round 2 results with top scoring subsections within each domain

Overall rank	Ranked principal domains Highest ranked subsection within each domain	Mean rank±SD	Mean rank of the 75% percentile of respondents	Weighted rank for subdomain
1*	**Good clinical care** Take and analyse a clinical and occupational history including an exposure history in a relevant, succinct and systematic manner	**2.8±2.1**	3.3	
		2.1±1.6		2.3
1*	**General principles of assessment and management of occupational hazards to health** Understand and apply the principles of risk assessment, that is, recognition of potential hazards in the work environment, evaluating risks and providing advice and information on control measures	**2.8±2.7**	3.5	
		2.3±2.1		2.2
3	**Assessment of disability and fitness for work** Assessing and advising on impairment, disability and fitness for work	**4.0±2.2**	4.3	
		1.5±1.3		3.0
4	**Communication skills** Be able to communicate effectively orally and in writing with patients and other stakeholders in a manner that they understand	**5.8±2.7**	6.7	
		1.7±1.4		5.3
5	**Ethical and legal issues** Be well informed about acts, regulations, codes of practice and guidance relevant to the workplace setting	**6.0±2.4**	6.7	
		2.0±1.3		11.2
6	**Team working and leadership skills** Understand how a team works effectively	**6.8±2.4**	7.5	
		2.2±1.4		15.7
7	**Environmental issues related to work practice** Recognise and advise on health risks in the general environment arising from industrial activities	**6.9±3.1**	8.4	
		2.1±1.4		18.0
8	**Health promotion** Assessing needs for health promotion in a workforce	**7.0±3.2**	8.4	
		1.4±0.6		20.6
9	**Clinical governance/clinical improvement** Practice evidence-based medicine	**7.3±2.3**	7.5	
		1.8±1.3		19.8
10	**Management skills** Be able to strategically plan and set objectives for delivering an occupational health service	**8.5±3.2**	10.0	
		2.3±1.3		24.5
11	**Teaching** and **educational supervision** Identify learning outcomes and construct educational objectives	**9.3±2.1**	9.2	
		2.6±2.1		27.9
12	**Research methods** Be able to define a problem in terms of needs for an evidence base	**10.7±2.0**	10.0	
		2.1±1.5		26.2

*Both domains are marked 1 because they were ranked the same.

Bold indicates the mean rank of the principal domains.

When items within a section have the same scores, this indicates that they were considered of the same priority.

In this second round, general principles of assessment and management of occupational hazards to health and good clinical care were jointly ranked as most important, followed by assessment of disability and fitness for work. Research methods was the domain considered least important, followed by teaching and educational supervision and management skills. Percentile calculations for the top three principal domains identified that 75% of respondents ranked good clinical care, general principles of assessment and management of occupational hazards to health and assessment of disability and fitness for work 3.3, 3.5 and 4.3 and higher in importance, respectively.

Table 3 also presents the top scoring subsection within each principal domain. Within many of the domains, that is, good clinical care, communication skills, assessment of disability and fitness for work, health promotion and legal and ethical issues, the top scoring subsections were what would be considered ‘core’ activities within those domains, including taking an appropriate clinical and occupational history and effective oral and written communication skills. Other top scoring subsections identified more specific priority areas. A detailed list of all subsections and their average and weighted ranks within each principal domain is presented in online supplementary table S2.

We tested for regional differences in the ranking of the principal domains within our sample using the Spearman’s rank test (see online supplementary table S3). From this, it is seen that the intercontinent rank comparisons are highly correlated, indicating no difference between European responses and those of other groups and no difference with the overall rank. Using the same correlation test, we performed subgroup analyses by age, gender and years of experience to investigate for possible differences. Our results indicate that the rankings were not statistically different at the 99% confidence level (see online supplementary tables S3–S6).

## Discussion

### Summary of findings

In this study, the views of specialist OPs on competency requirements have been sought from countries around the world. The UK survey also included OH nurses and those results will be presented in a separate publication.

By consensus, all the competency domains were regarded as important by respondents. The rating of all 12 identified domains was very high with scores of 90% and over in every domain. General principles of assessment and management of occupational hazards to health and good clinical care were jointly considered most important in ranking when compared with the other topic areas. For these two domains, 75% of respondents scored them high in priority with rank scores no higher than 3.5. In both rounds, research methods and teaching and educational supervision were considered least important. Management skills was ranked third lowest, which is surprising given its emerging role in OH practice, as reflected by the substantial proportion of ‘OP/manager’ respondents.

While subsection priorities reflected more predictable ‘core type’ activities in some principal domains, notably the domains of good clinical care and communication skills, it identified specific focus areas in other domains, for example in teaching and educational supervision and management skills.

Research methods ranked lowest overall as a principal domain, but its highest ranked subsection was being able to define a problem in terms of needs for an evidence base. This may suggest that, while respondents deemed an in-depth knowledge of research methods and direct involvement in research activities to be less of a priority, they acknowledge the importance of an evidence base in clinical practice. This is supported by the findings in round 2, where the highest subsection in the clinical governance/clinical improvement domain was practice evidence-based medicine.

General principles of assessment and management of occupational hazards to health ranked of highest priority could, at a first glance, suggest that OPs continue to harbour traditional ‘disease-focused’ views as mentioned in earlier studies.^13^ In contrast to the previous findings however, in this study, it now ‘shares’ the top spot with good clinical care. What has also emerged from respondent comments and feedback is that the definition of ‘occupational hazards to health’ has evolved over time and what it means and constitutes 15 years later has changed. There has been a shift from its original elements, for example, the identification of work-related ill health to other functions, for example, medical risk assessment, which includes assessing the impact of a specific health condition in relation to a particular role or working environment and advising (in addition to fitness for work) on associated short and longer term risks. This is increasingly becoming an important area of practice in many countries, including the UK.

Relatively new competencies such as team working and leadership skills have been identified as important in this study. This is likely to reflect the increasing multidisciplinary nature of OH practice, with the OP in many cases leader of these teams.

### Strengths and limitations

This is the first study to specifically establish current priorities among specialist OPs internationally of the common competencies required for OH practice. This has been derived from the opinions and experience of OPs working across a range of countries and sectors. It also incorporates perspectives from academics and managers. It has permitted participants from developed and developing countries to make an equal and independent contribution on their priorities towards identifying mutual global aims.

The list of competencies included in the questionnaire was taken from the training curricula of a range of OM institutions globally, to maximise the scope of the study. These curricula identified a high degree of crossover in terms of competency requirements between countries. Development of common core competencies has often been overshadowed by focus on differences in legislation among countries and varying ways in which the specialty is practised globally. This study has successfully established common priorities internationally, taking into account these variations.

Comments at the conference highlighted that, due to terminology and language variation among countries, certain items may have been interpreted in conceptually different ways. This could be considered a limitation. Measures to minimise this were implemented in both rounds through initial piloting of the questionnaires. Although English is widely used internationally, distribution of the questionnaire exclusively in this language may have limited potentially wider participation due to language barriers.

It proved difficult to get much response from Asian countries, notably China and Japan—important regions of rapid industrial development—which is a potential weakness of this study. Language barriers may have been a contributing factor. Furthermore, in both rounds, over 50% of responses were from the EU. While our analyses found no regional differences in opinion of our sample, the under-representation of some continents has limited the breadth of comparisons.

The intention originally was that all email distribution lists be copied to the study lead, in order that a response rate could be calculated. However, the pivotal role of professional networking media in distributing the questionnaire globally became evident during the first round. We therefore elected to distribute through this method as well. The result is that calculation of a response rate has not been possible, which could be a weakness. This approach, however, has enabled our survey to reach wider and even more remote parts of the world, thereby facilitating a more diverse and widely representative range of responses.

Comments from respondents highlighted that they found ranking somewhat more challenging than rating in having to commit to a priority. Although it is acknowledged that respondents may have considered some topics of equal importance, this requirement was necessary for the purpose of achieving priority consensus.

Addition of items from round 1 led to a longer but more comprehensive second questionnaire. However, no negative feedback was received around the length of time the questionnaire took to complete.

### Comparison with previous studies

The highest ranking domain is consistent with the earlier Macdonald *et al*^13^ European study where occupational hazards to health was the highest ranked principal domain. In Macdonald *et al,* however, research methods was considered a higher priority, ranked fourth. Law and ethics, although ranked fifth in our study, ranked second highest in Macdonald *et al* and was considered of highest priority in a study of UK customers’ views on required OH competencies.^15^ Assessment of disability and fitness for work and communication skills feature in the top five priorities in our and both those studies.^13^ ^15^ Owing to methodological and classification differences, it is difficult to make direct comparison with the Delclos study.^11^ The competency skill sets reported most commonly by respondents in the Delclos^11^ study were administrative/management (health and safety, legal, regulatory considerations), followed by professional practice (ethical considerations) and research.

## Conclusion

This study has established the current priorities among specialist OPs internationally of the common competencies required for OH practice. It has identified a high level of consensus among respondents on the identified competencies. These findings can be used to develop specialist OH training programmes and curricula in countries around the world. The results may also serve as a platform for the development of common core competencies/qualifications within specific geographical regions or, indeed, internationally. This is particularly pertinent with globalisation, the practice of OM across varying countries and free movement within the EU.

These mutually identified priorities can help to inform global policy on the delivery of OH services and, importantly, quality standards.
