# Supplementary material for: International perspective on common core competencies for occupational physicians: a modified Delphi study
Source: Occup Environ Med. 2016 Apr 13;73(7):452–8. doi: 10.1136/oemed-2015-103285 (PMC4941142; doi:10.1136/oemed-2015-103285)
Supplement: Supplementary data [file oemed-2015-103285supp.pdf]

Table S1. Ranking (Delphi-Round 2) Questionnaire for Occupational Physicians [distributed electronically using SmartSurvey]

STUDY OF THE COMPETENCIES OF OCCUPATIONAL PHYSICIANS (OP) WITHIN EUROPE AND GLOBALLY-ROUND 2-RANKING PHASE

1. By completing this questionnaire, you are consenting to take part in this entirely voluntary study

Please tick to confirm you have read the above statement

- [ ] Yes

2. You may receive this questionnaire several times- please only complete once.

Job title:

____

Country of practice:

____

Gender:

- [ ] Male
- [ ] Female

Age range:

- [ ] 18-24yrs
- [ ] 25-34yrs
- [ ] 35-44 yrs
- [ ] 45-54 yrs
- [ ] 55-64 yrs
- [ ] 65-74 yrs

Years of experience in specialty:

____

Which professional specialty body are you registered with or board certified by?

____

Main area of current OH practise:

- ☐ Healthcare
- ☐ Industry
- ☐ Academic
- ☐ Other

Specify

Are you a member of:

- ☐ EASOM (European Association of schools of Occupational Medicine)
- ☐ UEMS (Union of European Medical Specialities)

Did you complete a questionnaire in the first round of this study?
(please note, participation in the first round is not a requirement to take part in this questionnaire)

- ☐ Yes
- ☐ No

3. The following is a list of the elements Occupational Physicians are expected to be competent in when they complete their training. They comprise principle domains and then subsections. How important are they to you?

Please RANK them in order of importance using the scale ranges described at the start of each section. *No two items can be awarded the same score.*
Please note: Once you rank the first (and each subsequent item), the subsections will auto-adjust to list your rank choices in chronological order. This will enable you to check and review your choices before proceeding to the next domain.

A   <u>Good Clinical Care</u>
Please RANK each subsection in terms of their importance;

1= of most importance to 9 = of least importance

Broad general medical knowledge of all common medical conditions including diagnostic criteria, evidence based treatment spectrum and prognosis	
Take and analyse a clinical and occupational history including an exposure history in a relevant, succinct and systematic manner	
Perform a reliable and appropriate examination	
Interpret the results of investigations, including especially those relating to occupational attribution and functional prognosis	
Perform investigations competently where relevant	
Record concisely, accurately, confidentially and legibly all medical records, and date and sign all records	
Show empathy with and listen to employee.	
Appreciate the importance and interaction of psychological and social factors in patient's disease and illness behaviour.	

Manage time and problems effectively.

B General principles of assessment & management of occupational hazards

Please RANK each subsection in terms of their importance;

1= of most importance to 11 = of least importance

Understand and apply the principles of risk assessment- ie recognition of potential hazards in the work environment, evaluating risks and providing advice and information on control measures.
Provide advice on medical aspects/ factors relevant to the risk assessment.
Carry out a workplace visit and produce a report.
Understand the core principles of occupational/ industrial hygiene and be able to interpret reports and related measurements.
Understand the core principles of ergonomics and be able to interpret reports.
Understand the core principles of toxicology and be able to interpret reports and related measurements.
Identify work related ill health and provide advice on prognosis, prevention and management.
Carry out and evaluate health surveillance including biological monitoring for workers exposed to occupational hazards.
Evaluate and advise on first aid facilities in the workplace.
Advising on disaster and contingency planning within the organisation.
Understand the principles of Travel Medicine

C Assessment of disability and fitness for work.

Please RANK each subsection in terms of their importance;

1= of most importance to 10 = of least importance

Assessing and advising on impairment, disability and fitness for work
Advising on rehabilitation
Advising on redeployment
Assessing and advising on early retirement due to ill-health
Assessing and advising on sickness absence
Advising on legal issues related to disability
Advising on drug and alcohol problems
Assessing the ageing worker and advising on work ability
Advising on vocational rehabilitation
Liaising with other health professionals in assessing capability for work

D Environmental Issues Related to Work Practice

Please RANK each subsection in terms of their importance;

1= of most importance to 6 = of least importance

Recognise and advise on health risks in the general environment arising from industrial activities.
Assess and advise on the control of environmental exposures from the workplace.
Understand when to obtain environmental monitoring.
Interpret and explain the results of environmental monitoring.
Effectively communicate risk from various environmental exposures including water pollution, hazardous waste, sewage, household chemicals and radiation.
Identify and manage concerns about the health effects of human exposure to environmental hazards e.g. toxins and pollutants.

E Health Promotion

Please RANK each subsection in terms of their importance;

1= of most importance to 3 = of least importance

Assessing needs for health promotion in a workforce.
Give advice on nutritional and other healthy lifestyle issues.
Organising, providing and evaluating work related health promotion activities.

F Ethical and Legal issues

Please RANK each subsection in terms of their importance;

1= of most importance to 6 = of least importance

Be well-informed about acts, regulations, codes of practice and guidance relevant to the workplace setting.
Providing advice to managers, safety representatives and employees of their legal obligations.
Evaluate compliance with new legislation
Responsibilities relating to data protection.
Respect the patient's right to confidentiality.
Understand the process for gaining informed consent for clinical and research activities

G Clinical Governance/Clinical Improvement

Please RANK each subsection in terms of their importance;

1= of most importance to 6 = of least importance

Practise evidence based medicine.
Be able to handle and deal with complaints in a focused and constructive manner.
Develop and institute clinical guidelines and integrated care pathways. Be aware of advantages and disadvantages of guidelines.
Report and investigate critical incidents.
Be able to balance risk and benefits with patients.
Be actively involved in clinical audit.

H Communication skills

Please RANK each subsection in terms of their importance;

1= of most importance to 9 = of least importance

Be able to communicate effectively both orally and in writing to patients and other stakeholders in a manner that they understand
Prepare written reports on a range of topics for a range of groups including managers, unions (e.g. for safety representatives) and health professionals.
Demonstrate effective consultation skills.
Manage dissatisfied patients/ relatives.
Anticipate potential problems.
Be able to effectively participate in committees and to act as a chairperson.
Effective presentation skills. Make clear oral presentations to a range of audiences using audio-visual equipment.
Apply ethical principles when communicating with others about individuals.
Motivational interviewing skills

I Team working and leadership skills

Please RANK each subsection in terms of their importance;

1= of most importance to 6 = of least importance

Understand how a team works effectively
Be an effective team player
Understand different leadership styles
Demonstrate leadership
Delegation
Influencing and negotiation

J Teaching & Educational Supervision

Please RANK each subsection in terms of their importance;

1= of most importance to 9 = of least importance

Identify learning outcomes and construct educational objectives.
Design and deliver an effective teaching event or short course.
Teach large and small groups effectively.
Select and use appropriate teaching resources.
Give constructive effective feedback.
Evaluate programmes and events.
Use appropriate assessment methods.
Conduct effective appraisals.
Deliver effective mentorship

K Research methods

Please RANK each subsection in terms of their importance;

1= of most importance to 8 = of least importance

Be able to define a problem in terms of needs for an evidence base.

Be able to undertake a literature search.
Be able to undertake a systematic and critical appraisal of scientific literature.
Be able to develop and execute an appropriate study design
Be able to use databases.
Be able to carry out basic statistical analyses.
Present investigation and results in the format of a research based report.
To understand the principles of research ethics and ethical considerations in research.

L Management skills

Please RANK each subsection in terms of their importance;

1= of most importance to 8= of least importance

Be able to understand the principles and practice of management.
Be able to strategically plan and set objectives for delivering an occupational health service.
Finance management. Be able to negotiate and manage a budget/resources.
Evaluate the effectiveness and quality of an occupational health service.
Be able to market an occupational health service.
Define the roles and responsibilities of staff in providing an occupational health service
Support formulation of job descriptions.
Be able to manage a team.

Considering the above principle domain competencies and their subsections, we would ask you now to please RANK only the principle domains in order of importance using the scale 1 = of most importance and 12 = of least importance . Once again, no two items can be awarded the same score. *

Good Clinical Care
General principles of assessment & management of occupational hazards to health
Assessment of disability and fitness for work.
Environmental Issues Related to Work Practice
Health Promotion
Ethical and Legal issues
Clinical Governance/Clinical Improvement
Communication skills
Team working and leadership skills
Teaching & Educational Supervision
Research methods
Management skills

Table S2. Rank scores (mean, standardised mean,& weighted std rank) for all Principle domains and subsections

**Principle Domain**<br>Sub-domains	**Mean Rank score**	**Std Dev**	**Standardised Rank Score (StdRank)***	**Principle Domain Weight (-)**	**Weighted Standardised Rank (W_StdRank)****
**A. Good Clinical Care**					
A1. Broad general medical knowledge of all common medical conditions including diagnostic criteria, evidence based treatment spectrum and prognosis	3.2	2.4	3.5	1	3.5
A2. Take and analyse a clinical and occupational history including an exposure history in a relevant, succinct and systematic manner	2.1	1.6	2.3	1	2.3
A3. Perform a reliable and appropriate examination	4.3	2.0	4.7	1	4.7
A4. Interpret the results of investigations, including especially those relating to occupational attribution and functional prognosis	4.8	1.8	5.2	1	5.2
A5. Perform investigations competently where relevant	6.4	1.8	7.0	1	7.0
A6. Record concisely, accurately, confidentially and legibly all medical records, and date and sign all records	6.0	1.9	6.6	1	6.6
A7. Show empathy with and listen to employee.	5.1	2.4	5.6	1	5.6
A8. Appreciate the importance and interaction of psychological and social factors in patient's disease and illness behaviour.	5.6	2.3	6.1	1	6.1
A9. Manage time and problems effectively.	7.6	2.3	8.4	1	8.4
**B. General principles of assessment & management of occupational hazards to health**					
B1. Understand and apply the principles of risk assessment- ie recognition of potential hazards in the work environment, evaluating risks and providing advice and information on control measures.	2.3	2.1	2.2	1	2.2
B2. Provide advice on medical aspects/ factors relevant to the risk assessment.	3.5	2.1	3.3	1	3.3
B3. Carry out a workplace visit and produce a report.	4.7	2.5	4.3	1	4.3
B4. Understand the core principles of occupational/ industrial hygiene and be able to interpret reports and related measurements.	5.0	2.2	4.6	1	4.6
B5. Understand the core principles of ergonomics and be able to interpret reports.	6.1	1.8	5.6	1	5.6
B6. Understand the core principles of toxicology and be able to interpret reports and related measurements.	6.4	1.9	5.8	1	5.8
B7. Identify work related ill health and provide advice on prognosis, prevention and management.	4.0	2.6	3.7	1	3.7
B8. Carry out and evaluate health surveillance including biological monitoring for workers exposed to occupational hazards.	6.0	2.4	5.5	1	5.5
B9. Evaluate and advise on first aid facilities in the workplace.	8.9	1.7	8.1	1	8.1
B10. Advising on disaster and contingency planning within	9.3	1.7	8.5	1	8.5

the organisation.					
B11. Understand the principles of Travel Medicine	9.9	2.0	9.0	1	9.0
**C. Assessment of disability and fitness for work.**					
C1. Assessing and advising on impairment, disability and fitness for work	1.5	1.3	1.5	2	3.0
C2. Advising on rehabilitation	4.1	2.2	4.1	2	8.2
C3. Advising on redeployment	4.8	2.1	4.8	2	9.6
C4. Assessing and advising on early retirement due to ill-health	6.3	2.1	6.3	2	12.6
C5. Assessing and advising on sickness absence	4.6	2.4	4.6	2	9.2
C6. Advising on legal issues related to disability	6.8	2.3	6.8	2	13.7
C7. Advising on drug and alcohol problems	6.8	2.0	6.8	2	13.6
C8. Assessing the ageing worker and advising on work ability	6.6	2.5	6.6	2	13.2
C9. Advising on vocational rehabilitation	7.6	2.4	7.6	2	15.2
C10. Liaising with other health professionals in assessing capability for work	5.8	3.3	5.8	2	11.6
**D. Environmental Issues Related to Work Practice**					
D1. Recognise and advise on health risks in the general environment arising from industrial activities.	2.1	1.4	3.0	6	18.0
D2. Assess and advise on the control of environmental exposures from the workplace.	2.8	1.4	4.3	6	25.5
D3. Understand when to obtain environmental monitoring.	3.5	1.3	5.5	6	33.1
D4. Interpret and explain the results of environmental monitoring.	4.4	1.2	7.2	6	43.2
D5. Effectively communicate risk from various environmental exposures including water pollution, hazardous waste, sewage, household chemicals and radiation.	4.5	1.5	7.3	6	44.0
D6. Identify and manage concerns about the health effects of human exposure to environmental hazards eg. toxins and pollutants.	3.6	2.0	5.7	6	34.3
**E. Health Promotion**					
E1. Assessing needs for health promotion in a workforce.	1.4	0.6	2.9	7	20.6
E2. Give advice on nutritional and other healthy lifestyle issues.	2.5	0.7	7.8	7	54.9
E3. Organising, providing and evaluating work related health promotion activities.	2.0	0.7	5.7	7	40.0
**F. Ethical and Legal issues**					
F1. Be well-informed about acts, regulations, codes of practice and guidance relevant to the workplace setting.	2.0	1.3	2.8	4	11.2
F2. Providing advice to managers, safety representatives and employees of their legal obligations.	3.4	1.4	5.4	4	21.5
F3. Evaluate compliance with new legislation	4.4	1.3	7.1	4	28.6
F4. Responsibilities relating to data protection.	4.0	1.3	6.5	4	25.8
F5. Respect the patient's right to confidentiality.	2.4	1.5	3.6	4	14.3
F6. Understand the process for gaining informed consent for clinical and research activities	4.7	1.6	7.6	4	30.6
**G. Clinical Governance/Clinical Improvement**					
G1. Practise evidence based medicine.	1.8	1.3	2.5	8	19.8

G2. Be able to handle and deal with complaints in a focused and constructive manner.	3.5	1.5	5.4	8	43.3
G3. Develop and institute clinical guidelines and integrated care pathways. Be aware of advantages and disadvantages of guidelines.	3.3	1.5	5.2	8	41.4
G4. Report and investigate critical incidents.	3.9	1.2	6.3	8	50.4
G5. Be able to balance risk and benefits with patients.	3.4	1.6	5.4	8	43.1
G6. Be actively involved in clinical audit.	5.0	1.4	8.2	8	66.0
**H. Communication skills**					
H1. Be able to communicate effectively both orally and in writing to patients and other stakeholders in a manner that they understand	1.7	1.4	1.8	3	5.3
H2. Prepare written reports on a range of topics for a range of groups including managers, unions (e.g. for safety representatives) and health professionals.	4.0	2.0	4.4	3	13.3
H3. Demonstrate effective consultation skills.	3.8	1.9	4.2	3	12.6
H4. Manage dissatisfied patients/ relatives.	5.7	1.7	6.3	3	19.0
H5. Anticipate potential problems.	5.0	1.9	5.5	3	16.4
H6. Be able to effectively participate in committees and to act as a chairperson.	6.5	1.8	7.2	3	21.6
H7. Effective presentation skills. Make clear oral presentations to a range of audiences using audio-visual equipment.	6.3	1.9	7.0	3	21.0
H8. Apply ethical principles when communicating with others about individuals.	4.4	2.7	4.8	3	14.4
H9. Motivational interviewing skills	7.5	2.2	8.3	3	24.9
**I. Team working and leadership skills**					
I1. Understand how a team works effectively	2.2	1.4	3.1	5	15.7
I2. Be an effective team player	2.5	1.4	3.8	5	18.8
I3. Understand different leadership styles	3.7	1.5	5.8	5	28.9
I4. Demonstrate leadership	3.5	1.5	5.5	5	27.3
I5. Delegation	4.7	1.2	7.7	5	38.5
I6. Influencing and negotiation	4.4	1.7	7.2	5	35.8
**J. Teaching & Educational Supervision**					
J1. Identify learning outcomes and construct educational objectives.	2.6	2.1	2.8	10	27.9
J2. Design and deliver an effective teaching event or short course.	3.7	2.2	4.1	10	40.6
J3.Teach large and small groups effectively.	4.3	2.1	4.8	10	47.5
J4. Select and use appropriate teaching resources.	4.4	1.9	4.8	10	47.9
J5. Give constructive effective feedback.	4.6	1.9	5.0	10	50.0
J6. Evaluate programmes and events.	6.4	2.1	7.1	10	71.2
J7. Use appropriate assessment methods.	6.0	2.0	6.6	10	66.4
J8. Conduct effective appraisals.	6.8	2.3	7.5	10	75.0
J9. Deliver effective mentorship	6.2	3.1	6.8	10	68.3
**K. Research methods**					
K1. Be able to define a problem in terms of needs for an evidence base.	2.1	1.5	2.4	11	26.2

K2. Be able to undertake a literature search.	2.8	1.5	3.3	11	36.7
K3. Be able to undertake a systematic and critical appraisal of scientific literature.	3.2	1.5	3.8	11	42.2
K4. Be able to develop and execute an appropriate study design	4.4	1.5	5.4	11	58.9
K5. Be able to use databases.	5.3	1.6	6.5	11	71.7
K6. Be able to carry out basic statistical analyses.	6.2	1.5	7.7	11	84.2
K7. Present investigation and results in the format of a research based report.	6.7	1.4	8.3	11	91.7
K8. To understand the principles of research ethics and ethical considerations in research.	5.3	2.6	6.6	11	72.2
**L. Management skills**					
L1. Be able to understand the principles and practice of management.	2.8	2.0	3.3	9	29.8
L2. Be able to strategically plan and set objectives for delivering an occupational health service.	2.3	1.3	2.7	9	24.5
L3. Finance management. Be able to negotiate and manage a budget/resources.	5.3	1.7	6.5	9	58.3
L4. Evaluate the effectiveness and quality of an occupational health service.	4.0	1.7	4.9	9	44.1
L5. Be able to market an occupational health service.	6.0	1.7	7.4	9	67.0
L6. Define the roles and responsibilities of staff in providing an occupational health service	4.6	1.7	5.6	9	50.6
L7. Support formulation of job descriptions.	7.0	1.4	8.7	9	78.1
L8. Be able to manage a team.	4.0	2.5	4.8	9	43.6
**M. Principle Domains**					
M1. Good Clinical Care	2.8	2.7	2.5		
M2. General principles of assessment & management of occupational hazards to health	2.8	2.1	2.5		
M3. Assessment of disability and fitness for work.	4.0	2.2	3.5		
M4. Environmental Issues Related to Work Practice	6.9	3.1	5.8		
M5. Health Promotion	7.0	3.2	5.9		
M6. Ethical and Legal issues	6.0	2.4	5.1		
M7. Clinical Governance/Clinical Improvement	7.3	2.3	6.1		
M8. Communication skills	5.8	2.7	4.9		
M9. Team working and leadership skills	6.8	2.4	5.8		
M10.Teaching & Educational Supervision	9.3	2.1	7.8		
M11. Research methods	10.7	2.0	8.9		
M12. Management skills	8.5	3.2	7.2		

*Standardised Ranks (StdRank) Score to a scale of 1-10 using the formula: *9*((Average Rank-1)/(No. of categories-1))+1*
** Weighted Standardise Rank (W_StdRank) = Weight of ranked Principle Domain * StdRank

Table S3. Inter-continental (all respondents, EU respondent, Americas: North & South America; and all other countries) comparison of the Principle Domain ranking order

	All_Mean Rank	All_Rank	EU	EU_Rank	Americas	Americas_Rank	Other	Other_Rank
**M. Principle Domains**								
M1. Good Clinical Care	**2.8**	**1**	2.6	**1**	2.5	**1**	4.2	**3**
M2. General principles of assessment & management of occupational hazards to health	2.8	**1**	3.2	**2**	2.7	**2**	1.9	**1**
M3. Assessment of disability and fitness for work.	4.0	**3**	3.8	**3**	4.5	**3**	4.0	**2**
M4. Environmental Issues Related to Work Practice	6.9	**7**	8.0	**9**	6.0	**4**	5.3	**4**
M5. Health Promotion	7.0	**8**	7.8	**8**	6.2	**6**	6.6	**8**
M6. Ethical and Legal issues	6.0	**5**	5.9	**5**	6.0	**4**	6.3	**5**
M7. Clinical Governance/Clinical Improvement	7.3	**9**	6.7	**7**	7.6	**9**	8.3	**9**
M8. Communication skills	5.8	**4**	5.3	**4**	6.2	**6**	6.4	**6**
M9. Team working and leadership skills	6.8	**6**	6.6	**6**	7.3	**8**	6.5	**7**
M10.Teaching & Educational Supervision	9.3	**11**	9.3	**11**	9.4	**11**	9.0	**11**
M11. Research methods	10.7	**12**	10.7	**12**	10.8	**12**	10.5	**12**
M12. Management skills	8.5	**10**	8.1	**10**	9.0	**10**	8.9	**10**

Spearmans' Rank Correlation	Rho	*p*
All versus EU Rank	0.97	<0.01
All versus Americas Rank	0.93	<0.01
All versus Other Rank	0.94	<0.01
EU versus Americas Rank	0.87	<0.01
EU versus Other Rank	0.86	<0.01
Americas versus Others Rank	0.96	<0.01

Table S4. Age subgroup comparison of the Principle Domain ranking order

	All (Mean Rank)	All_Rank	25-35 years (Mean Rank)	25-34_Rank	35-44 years (Mean Rank)	35-44_Rank	45-54 years (Mean Rank)	45-54_Rank	55-64 years (Mean Rank)	55-64_rank	65-74 years (Mean Rank)	65-74_Rank
**M. Principle Domains**												
M1. Good Clinical Care	2.8	**1**	2.5	**1**	2.4	**1**	2.8	**1**	3.1	**2**	2.9	**2**
M2. General principles of assessment & management of occupational hazards to health	**2.8**	**1**	2.5	**1**	3.3	**2**	2.8	**1**	2.7	**1**	2.4	**1**
M3. Assessment of disability and fitness for work.	4.0	**2**	3.5	**2**	4.3	**3**	4.0	**2**	4.0	**3**	3.8	**3**
M4. Environmental Issues Related to Work Practice	6.9	**6**	5.8	**5**	7.2	**7**	7.3	**6**	6.7	**6**	6.0	**4**
M5. Health Promotion	7.0	**7**	4.0	**3**	5.9	**5**	7.5	**7**	7.1	**9**	8.6	**10**
M6. Ethical and Legal issues	6.0	**4**	8.3	**7**	5.9	**5**	5.8	**3**	6.1	**5**	6.1	**5**
M7. Clinical Governance/Clinical Improvement	7.3	**8**	9.0	**9**	7.6	**8**	7.3	**6**	7.0	**8**	7.4	**8**
M8. Communication skills	5.8	**3**	7.3	**6**	5.7	**4**	6.0	**4**	5.5	**4**	6.4	**6**
M9. Team working and leadership skills	6.8	**5**	5.5	**4**	7.0	**6**	6.7	**5**	6.8	**7**	7.1	**7**
M10.Teaching & Educational Supervision	9.3	**10**	8.8	**8**	9.2	**10**	8.9	**9**	9.6	**11**	9.1	**11**
M11. Research methods	10.7	**11**	10.5	**10**	10.6	**11**	10.8	**10**	10.7	**12**	10.5	**12**
M12. Management skills	8.5	**9**	10.5	**11**	8.8	**9**	8.2	**8**	8.7	**10**	7.8	**9**

**Spearmans' Rank Correlation**	**Rho**	***p***
All vs 25-34	0.84	<0.01
All vs 35-44	0.97	<0.01
All vs 45-54	0.98	<0.01
All vs 55-64	0.98	<0.01
All vs 65-74	0.93	<0.01

Table S5. Gender comparison of the Principle Domain ranking order

	All (Mean Rank)	All_Rank	Male (Mean Rank)	Male_Rank	Female (Mean Rank)	Female_Rank
**M. Principle Domains**						
M1. Good Clinical Care	**2.8**	**1**	2.9	**2**	2.7	**1**
M2. General principles of assessment & management of occupational hazards to health	2.8	**1**	2.6	**1**	3.2	**2**
M3. Assessment of disability and fitness for work.	4.0	**2**	4.1	**3**	4.0	**3**
M4. Environmental Issues Related to Work Practice	6.9	**6**	6.8	**5**	7.0	**8**
M5. Health Promotion	7.0	**7**	7.2	**7**	6.7	**6**
M6. Ethical and Legal issues	6.0	**4**	5.9	**4**	6.2	**5**
M7. Clinical Governance/Clinical Improvement	7.3	**8**	7.4	**8**	7.1	**9**
M8. Communication skills	5.8	**3**	5.9	**4**	5.6	**4**
M9. Team working and leadership skills	6.8	**5**	6.9	**6**	6.8	**7**
M10.Teaching & Educational Supervision	9.3	**10**	9.3	**10**	9.3	**11**
M11. Research methods	10.7	**11**	10.8	**11**	10.4	**12**
M12. Management skills	8.5	**9**	8.3	**9**	9.0	**10**

**Spearmans' Rank Correlation**	**Rho**	***p***
All versus Male	0.99	<0.01
All versus Female	0.98	<0.01
Male versus	0.96	<0.01

Table S6. Years of Experience as Occupational Physician (under 25 years; over 25 years) comparison of the Principle Domain ranking order

	**All (Mean Rank)**	**All_Rank**	**Experience_ Under25years (Mean Rank)**	**Experience_ Under25years_ Rank**	**Experience_ Over25years (Mean Rank)**	**Experience_ Over25years_ Rank**
**M. Principle Domains**						
M1. Good Clinical Care	2.8	**1**	2.6	**1**	3.1	**2**
M2. General principles of assessment & management of occupational hazards to health	**2.8**	**1**	3.0	**2**	2.5	**1**
M3. Assessment of disability and fitness for work.	4.0	**2**	4.0	**3**	4.1	**3**
M4. Environmental Issues Related to Work Practice	6.9	**6**	7.1	**8**	6.6	**6**
M5. Health Promotion	7.0	**7**	6.6	**6**	7.8	**9**
M6. Ethical and Legal issues	6.0	**4**	6.1	**5**	5.9	**5**
M7. Clinical Governance/Clinical Improvement	7.3	**8**	7.5	**9**	7.0	**8**
M8. Communication skills	5.8	**3**	5.8	**4**	5.7	**4**
M9. Team working and leadership skills	6.8	**5**	6.8	**7**	6.9	**7**
M10.Teaching & Educational Supervision	9.3	**10**	9.3	**11**	9.2	**11**
M11. Research methods	10.7	**11**	10.7	**12**	10.7	**12**
M12. Management skills	8.5	**9**	8.5	**10**	8.5	**10**

**Spearmans' Rank Correlation**	**Rho**	***p***
All vs Under 25 years experience	0.98	<0.01
All vs Over 25 years experience	0.98	<0.01
Under vs Over 25 years experience	0.94	<0.01
